# Phase I study of ipilimumab in phased combination with paclitaxel and carboplatin in Japanese patients with non-small-cell lung cancer

**DOI:** 10.1007/s10637-015-0243-5

**Published:** 2015-05-01

**Authors:** Hidehito Horinouchi, Noboru Yamamoto, Yutaka Fujiwara, Ikuo Sekine, Hiroshi Nokihara, Kaoru Kubota, Shintaro Kanda, Shigehiro Yagishita, Hiroshi Wakui, Satoru Kitazono, Hidenori Mizugaki, Takuto Tokudome, Tomohide Tamura

**Affiliations:** Department of Thoracic Oncology, National Cancer Center Hospital, Tsukiji 5-1-1, Chuo-ku, Tokyo 104-0045 Japan; Bristol-Myers K.K., Clinical Research, R&D, 6-5-1 Nishishinjyuku, Shinjyuku-ku, Tokyo 163-1328 Japan

**Keywords:** Ipilimumab, Paclitaxel, Carboplatin, Non-small-cell lung cancer, Phase I study, Japanese patients

## Abstract

*Background* Ipilimumab is an antibody that targets the cytotoxic T-lymphocyte antigen-4 to potentiate an antitumor response. Adding ipilimumab 10 mg/kg to paclitaxel (PTX) and carboplatin (CBDCA) in a phased schedule improved progression-free survival in a phase II non-small-cell lung cancer (NSCLC) study. *Methods* This dose-escalating, phase I study was designed to identify the recommended dose of ipilimumab (3 or 10 mg/kg) by evaluating dose-limiting toxicity (DLT; Cycles 3 and 4) in phased combination with PTX (175 mg/m^2^) and CBDCA (area under the curve = 6) in Japanese patients with advanced NSCLC. Treatment was administered intravenously every 3 weeks initially, followed by some eligible patients receiving maintenance ipilimumab once every 12 weeks. Additional endpoints included safety, tumor response, pharmacokinetics, and immunogenicity. *Results* Fifteen patients were enrolled and 12 received ipilimumab (*n* = 6, 3 mg/kg; *n* = 6, 10 mg/kg) in combination with PTX and CBDCA. DLTs occurred in 2 patients (ipilimumab 3 mg/kg) and 1 patient (ipilimumab 10 mg/kg). The most common grade 3/4 adverse events (AEs) were decreased hemoglobin, leukopenia, and neutropenia. The most common immune-related AEs affected the skin, gastrointestinal, and nervous system. The safety profile was similar in both cohorts. Three patients in each cohort achieved a partial response. The pharmacokinetic (PK) profile of ipilimumab in Japanese patients was similar to that observed in previous studies in non-Japanese patients. *Conclusions* The recommended dose of ipilimumab in phased combination with PTX and CBDCA in Japanese patients with NSCLC was identified as 10 mg/kg. The safety profile was consistent with the previously defined AE profile.

## Introduction

Patients with newly diagnosed non-small-cell lung cancer (NSCLC) typically present with locally advanced stage III or metastatic stage IV disease. Platinum-based chemotherapy combinations, the current standard of care for this patient population, offer responses of between 30 and 60 %, but survival benefit is limited (median overall survival of approximately 9 months), and new treatment options are needed [1]. One of the most promising developments in the search for novel treatments has been the advent of immune-checkpoint inhibitors that harness the power of the patient’s own immune system to fight cancer [2]. Ipilimumab is a fully human, monoclonal antibody that targets the immune-checkpoint cytotoxic T-lymphocyte antigen-4 (CTLA-4) to potentiate T-cell-mediated antitumor response [3]. Ipilimumab is approved as either first- or second-line therapy in over 40 countries for the treatment of unresectable or metastatic melanoma, but is not currently approved in Japan [4, 5]. The potential use of ipilimumab in the treatment of other tumor types is an area of intense investigation.

Recently, ipilimumab 10 mg/kg in combination with paclitaxel (PTX) and carboplatin (CBDCA) was evaluated in patients with previously untreated, advanced NSCLC in a randomized, multicenter, double blind, phase II study [6]. In this study, patients received ipilimumab in a phased or concurrent regimen. Patients treated with the phased regimen received two doses of placebo plus PTX/CBDCA, followed by four doses of ipilimumab plus PTX/CBDCA; those treated with the concurrent regimen received four doses of ipilimumab plus PTX/CBDCA, followed by two doses of placebo plus PTX/CBDCA. Treatment was administered intravenously every 3 weeks for ≤18 weeks (induction). Eligible patients continued ipilimumab or placebo every 12 weeks as maintenance therapy. The addition of ipilimumab to PTX/CBDCA in a phased schedule significantly increased progression-free survival (PFS) compared with PTX/CBDCA alone. No statistically significant difference was observed between concurrent ipilimumab and PTX/CBDCA versus PTX/CBDCA alone. Ipilimumab in combination with PTX/CBDCA was well tolerated. Taken together, these results suggest that ipilimumab in combination with PTX/CBDCA on a phased schedule was safe and active in this patient population.

The primary objective of this open-label, dose-escalation, phase I study (CA184-113, ClinicalTrials.gov: NCT01165216) was to establish the recommended dose of ipilimumab in a phased combination regimen with PTX/CBDCA in Japanese patients with advanced or metastatic NSCLC. Safety, tumor response, pharmacokinetics, and immunogenicity were also assessed.

## Methods

### Patients

Japanese patients of either sex were eligible to participate in this study if they had histologically- or cytologically-documented stage IIIB NSCLC and were not candidates for definitive thoracic radiotherapy, stage IV NSCLC, or recurrent disease following radiotherapy and/or surgery. Eligible patients were required to have adequate bone marrow function (hemoglobin ≥ 9.0 g/dL, absolute neutrophil count [ANC] ≥ 1500/mm^3^, and platelet count ≥ 100,000/mm^3^), liver function (total bilirubin ≤ 2.0 times the upper limit of normal [x ULN], aspartate aminotransferase [AST] ≤ 2.5 × ULN, alanine aminotransferase [ALT] ≤ 2.5 × ULN), and renal function (calculated creatinine clearance based on Cockcroft and Gault formula ≥ 50 mL/min). Other inclusion criteria included age of at least 20 years; no prior chemotherapy, hormonal therapy, immunotherapy or targeted therapy for NSCLC; a life expectancy of at least 3 months; an Eastern Cooperative Oncology Group (ECOG) performance status of 0 or 1; and the ability and willingness to comply with visits and procedures as specified in the protocol (patients were admitted to the hospital from at least Day 1 to Day 22 of the third treatment cycle).

Patients were ineligible to participate in this study if they had symptomatic brain metastases or brain metastases requiring medication, a history of autoimmune disease or motor neuropathy of autoimmune origin, peripheral neuropathy of grade 2 or higher, diarrhea of grade 2 or higher, current or a history of gastrointestinal perforations, and chronic use of immunosuppressants and/or systemic corticosteroids.

### Study oversight

This study was sponsored by Bristol-Myers K.K. All procedures performed in studies involving human participants were in accordance with the ethical standards of the institutional and/or national research committee and with the 1964 Helsinki declaration and its later amendments or comparable ethical standards. The study protocol was approved by the Institutional Review Board and research was conducted in accordance with the standards specified by Article 14 Paragraph 3 and Article 80-2 of the Pharmaceutical Affairs Law, and Good Clinical Practice, as defined by the Ministerial Ordinance Concerning the Standards for the Implementation of Clinical Studies on Pharmaceutical Products and concerning notifications. All study participants provided written informed consent prior to enrollment.

### Study design and treatment

**Fig. 1:**
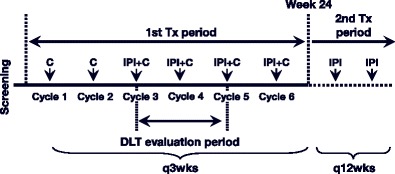
Schema for study CA184-113. C, paclitaxel/carboplatin; DLT, dose-limiting toxicity; IPI, ipilimumab; Tx, treatment; q3wks, every 3 weeks; q12wks, every 12 weeks

In this open-label, dose-escalation, phase I study, patients with advanced or metastatic NSCLC were treated with ipilimumab (3 or 10 mg/kg) in combination with PTX (175 mg/m^2^) and CBDCA (area under the curve [AUC] = 6). The treatment schedule was divided into two treatment periods (Fig. 1). During the first treatment period, patients received phased ipilimumab, which consisted of two cycles of PTX/CBDCA every 3 weeks (q3wks) followed by four cycles of ipilimumab plus PTX/CBDCA q3wks. During the second treatment period, patients without disease progression received ipilimumab alone once every 12 weeks (q12wks) as maintenance treatment. To be eligible for maintenance treatment, patients were required to meet core safety criteria and have adequate bone marrow function (ANC > 1500/mm^3^ and platelet count > 100,000/mm^3^). Patients were enrolled in successive cohorts of 3 to 6 patients using a standard ‘3 + 3’ design. The recommended dose was defined as the highest dose at which not more than 2 out of 6 patients experienced a dose-limiting toxicity (DLT), taking into consideration the profile of DLTs and the frequency and severity of toxicities after the DLT evaluation period. The final recommended dose was agreed upon by the Sponsor and principal investigator. Patients received ipilimumab at 3 mg/kg (Cohort A) or 10 mg/kg (Cohort B).

### Study endpoints and assessments

The primary objective of this study was to identify the recommended dose of ipilimumab in a phased combination regimen with PTX/CBDCA in Japanese patients with advanced or metastatic NSCLC. The recommended dose was defined as the highest dose at which not more than 2 out of 6 patients had DLT during the first two cycles after ipilimumab treatment (i.e., from Day 1 at Cycle 3 to Day 21 at Cycle 4). Dose-limiting toxicities included any of the following: grade 4 ANC decrease lasting for 7 days or more; febrile neutropenia lasting more than 3 days; grade 4 platelet count decrease; grade 3 platelet count decrease requiring transfusion; grade 3 or higher nausea, vomiting, and/or diarrhea despite the use of adequate/maximal medical intervention; grade 3 or higher AST/ALT levels not resolved to grade 2 or less within 2 weeks of onset; grade 3 or higher rash not resolved to grade 2 or less within 2 weeks of onset; and any grade 3 or higher non-hematological toxicity (except grade 3 fatigue, grade 3 asthenia, grade 3 transient arthralgia/myalgia, and grade 3 transient abnormal electrolyte).

Secondary objectives were safety and tolerability, tumor response, and pharmacokinetics (PK) of ipilimumab. Safety and tolerability was defined as the incidence of AEs occurring throughout the study period. These assessments were based on the National Cancer Institute Common Terminology Criteria for Adverse Events (NCI-CTCAE) version 3.0 [7]. Tumor response was defined as the best overall response rate (BORR). Responses were based on Response Evaluation Criteria in Solid Tumors (RECIST) version 1.1 [8]. Blood serum samples for assessing PK of ipilimumab were drawn at the following time points: Cycle 3 (Day 1, 2, 3, 8, and 15), Cycle 4 (Day 1), Cycle 5 (Day 1), Cycle 6 (Day 1), Week 24 and subsequent cycles, and off treatment. Quantification of ipilimumab in serum was performed using a validated enzyme linked immunosorbent assay (ELISA). Ipilimumab PKs were derived from serum concentration-versus-time data. Pharmacokinetic parameters included peak plasma concentration (C_max_), area under the concentration-versus-time curve from time zero to Day 21 (AUC_0-21d_), time of maximum observed serum concentration (T_max_), serum half-life (T-HALF), clearance (CL), and volume of distribution at steady-state (Vss). Geometric mean and coefficient of variation were calculated for C_max_, AUC_0-21d_, CL, and Vss. Mean and standard deviation were calculated for T-HALF. Median, minimum and maximum were calculated for T_max_.

Immunogenicity was evaluated as an exploratory endpoint. Blood samples were evaluated for the presence of antibodies to ipilimumab (human anti-human antibodies [HAHA]). Blood samples were drawn pre-dose on Day 1 of the third cycle and prior to each subsequent dose of ipilimumab, off treatment, and at first follow-up in case of discontinuation before Week 24. The detection of HAHA was performed using a validated bridging electrochemiluminescence immunoassay, which provided a semi-quantitative assessment of immunogenicity for HAHA using pre-established cut-points.

## Results

### Patients and treatment

Fifteen patients with advanced or metastatic NSCLC were enrolled and treated at the National Cancer Center Hospital in Tokyo, Japan between September 2010 and June 2013; patient characteristics at baseline are provided in Table 1. Eight patients were enrolled in Cohort A (ipilimumab 3 mg/kg), and 7 patients were enrolled in Cohort B (ipilimumab 10 mg/kg). Six patients in each study cohort received ipilimumab treatment. Three patients were discontinued prior to receiving ipilimumab. The reasons for discontinuation were intolerable toxicity (*n* = 2, Cohort A) and withdrawal of informed consent (*n* = 1, Cohort B). The median age was similar in the two dose cohorts and the majority of patients were male with stage IV disease. All patients had adenocarcinoma, with the exception of one patient in Cohort B who had squamous cell carcinoma.

**Table 1:** Patient characteristics

Characteristic	Cohort A	Cohort B	Total (*N* = 15^a^)
	(*n* = 8)	(*n* = 7)	
	Ipi 3 mg/kg	Ipi 10 mg/kg	
Number of ipilimumab-treated patients	6	6	12
Sex, n (%)			
Male	6 (75.0)	6 (85.7)	12 (80.0)
Female	2 (25.0)	1 (14.3)	3 (20.0)
Age, years			
Median	61.5	61.0	61.0
Range	27–65	40–70	27–70
Disease status, n (%)			
Stage IV	6 (75.0)	5 (71.4)	11 (73.3)
Recurrent	2 (25.0)	2 (28.6)	4 (26.7)
Tumor type, n (%)			
Adenocarcinoma	8 (100.0)	6 (85.7)	14 (93.3)
Squamous cell carcinoma	0 (0.0)	1 (14.3)	1 (6.7)

*Ipi* ipilimumab

^a^Including patients who were discontinued before receiving ipilimumab due to intolerable toxicity associated with chemotherapy (*n* = 2, Cohort A) and withdrawal of informed consent (*n* = 1, Cohort B)

Treatment and dosing information is provided in Table 2. The majority of patients required a dose delay of study drug (*n* = 11/12, ipilimumab; *n* = 12/15, PTX/CBDCA). Two patients in Cohort A (ipilimumab 3 mg/kg) and one patient in Cohort B (ipilimumab 10 mg/kg) required a chemotherapy dose reduction, which occurred during the fifth or sixth treatment cycle. One patient in Cohort B received a maintenance dose of ipilimumab, and all patients were off treatment at the end of the study. For the total study population, the reasons for study discontinuation (*n* = 15) were study drug toxicity (60 %), disease progression (26.7 %), and discontinuation at the patient’s request (13.3 %). In Cohort A (ipilimumab 3 mg/kg, *n* = 8), 6 patients discontinued due to study drug toxicity (75 %), 1 patient discontinued due to disease progression (12.5 %), and 1 patient requested to discontinue (12.5 %). In Cohort B (ipilimumab 10 mg/kg, *n* = 7), 3 patients discontinued due to study drug toxicity (42.9 %), 3 patients discontinued due to disease progression (42.9 %), and 1 patient requested to discontinue (14.3 %).

**Table 2:** Treatment and number of doses that patients received

	Cohort A	Cohort B	Total (*N* = 15)
	(*n* = 8)	(*n* = 7)	
	Ipi 3 mg/kg	Ipi 10 mg/kg	
Number of ipilimumab-treated patients, n	6	6	12
Number of doses, n			
Ipilimumab			
0	2 (25.0)	1 (14.3)	3 (20.0)
1	0 (0)	0 (0)	0 (0)
2	3 (37.5)	1 (14.3)	4 (26.7)
3	0 (0)	2 (28.6)	2 (13.3)
4	3 (37.5)	2 (28.6)	5 (33.3)
5 (maintenance dose)	0 (0)	1 (14.3)	1 (6.7)
6 (maintenance dose)	0 (0)	0 (0)	0 (0)
Paclitaxel/carboplatin			
0	0 (0)	0 (0)	0 (0)
1	2 (25.0)	0 (0)	2 (13.3)
2	0 (0)	1 (14.3)	1 (6.7)
3	0 (0)	0 (0)	0 (0)
4	3 (37.5)	1 (14.3)	4 (26.7)
5	0 (0)	4 (57.1)	4 (26.7)
6	3 (37.5)	1 (14.3)	4 (26.7)

*Ipi* ipilimumab

### Safety data

In Cohort A (ipilimumab 3 mg/kg), DLT was reported in 2 of 6 patients. One patient experienced grade 3 febrile neutropenia and grade 4 increased amylase after 4 doses of chemotherapy and 2 doses of ipilimumab. The febrile neutropenia resolved after treatment with granulocyte colony-stimulating factor (G-CSF) and antibiotics; the increased amylase required no treatment. Another patient experienced grade 4 thrombocytopenia after 4 doses of chemotherapy and 2 doses of ipilimumab. The thrombocytopenia resolved after a platelet transfusion. In Cohort B (ipilimumab 10 mg/kg), DLT was reported in 1 of 6 patients. This patient experienced grade 3 enterocolitis, grade 3 increased total bilirubin, and grade 4 increased lipase after 4 doses of chemotherapy and two doses of ipilimumab. The enterocolitis and increased total bilirubin resolved with treatment, which included a high-dose steroid (prednisone), levofloxacin, meropenem, and parenteral nutrition; and the increase lipase resolved without treatment.

A summary of treatment-related adverse events (AEs) in patients who received PTX/CBDCA and at least one dose of ipilimumab is shown in Table 3. The most common grade 3/4 AEs (occurring in 3 or more patients) were neutropenia (*n* = 6, Cohort A; *n* = 6, Cohort B), decreased hemoglobin (*n* = 4, Cohort B), and leukopenia (*n* = 3, Cohort A; *n* = 1, Cohort B). The most frequent lower-grade (1/2) AEs that occurred in greater than 20 % of patients in either cohort included decreased appetite (*n* = 4, cohort A; *n* = 5, cohort B), arthralgia (*n* = 3, cohort A, *n* = 5, cohort B), rash (*n* = 5, cohort A; *n* = 3, cohort B), nausea (*n* = 4, cohort A; *n* = 3, cohort B), prolonged QT (*n* = 4, cohort A; *n* = 2, cohort B), fatigue (*n* = 2, cohort A; *n* = 4, cohort B), and peripheral sensory neuropathy (*n* = 1, cohort A; *n* = 5, cohort B).

**Table 3:** Summary of treatment-related adverse events in patients who received PTX/CBDCA and at least one dose of ipilimumab

Toxicities^a,b^	Cohort A			Cohort B		
	(*n* = 6)			(*n* = 6)		
	Ipi 3 mg/kg			Ipi 10 mg/kg		
	Total (G1-4)	G3	G4	Total (G1-4)	G3	G4
Hematological, n (%)						
Hemoglobin decreased	0 (0)	0 (0)	0 (0)	4 (66.7)	4 (66.7)	0 (0)
Leukopenia	3 (50.0)	3 (50.0)	0 (0)	1 (16.7)	1 (16.7)	0 (0)
Lymphopenia	1 (16.7)	0 (0)	1 (16.7)	0 (0)	0 (0)	0 (0)
Neutropenia	6 (100.0)	0 (0)	6 (100.0)	6 (100.0)	3 (50.0)	3 (50.0)
Febrile neutropenia	1 (16.7)	1 (16.7)	0 (0)	0 (0)	0 (0)	0 (0)
Thrombocytopenia	1 (16.7)	0 (0)	1 (16.7)	2 (33.3)	0 (0)	0 (0)
Gastrointestinal, n (%)						
Nausea	4 (66.7)	0 (0)	0 (0)	3 (50.0)	0 (0)	0 (0)
Diarrhea	3 (50.0)	0 (0)	0 (0)	1 (16.7)	0 (0)	0 (0)
Constipation	2 (33.3)	0 (0)	0 (0)	2 (33.3)	0 (0)	0 (0)
Vomiting	1 (16.7)	0 (0)	0 (0)	2 (33.3)	0 (0)	0 (0)
Enterocolitis	0 (0)	0 (0)	0 (0)	1 (16.7)	1 (16.7)	0 (0)
Metabolism and nutrition disorders, n (%)						
Decreased appetite	4 (66.7)	0 (0)	0 (0)	5 (83.3)	0 (0)	0 (0)
Hypocalcaemia	1 (16.7)	1 (16.7)	0 (0)	0 (0)	0 (0)	0 (0)
Hypomagnesaemia	1 (16.7)	0 (0)	1 (16.7)	0 (0)	0 (0)	0 (0)
Dermatologic, n (%)						
Rash	5 (83.3)	0 (0)	0 (0)	3 (50.0)	0 (0)	0 (0)
Alopecia	1 (16.7)	0 (0)	0 (0)	2 (33.3)	0 (0)	0 (0)
Dry skin	1 (16.7)	0 (0)	0 (0)	2 (33.3)	0 (0)	0 (0)
Pruritus	0 (0)	0 (0)	0 (0)	2 (33.3)	0 (0)	0 (0)
Mediastinal disorders, n (%)						
Hiccups	0 (0)	0 (0)	0 (0)	2 (33.3)	0 (0)	0 (0)
Oropharyngeal pain	2 (33.3)	0 (0)	0 (0)	2 (33.3)	0 (0)	0 (0)
Musculoskeletal and connective tissue disorders, n (%)						
Arthralgia	3 (50.0)	0 (0)	0 (0)	5 (83.3)	0 (0)	0 (0)
Peripheral sensory neuropathy	1 (16.7)	0 (0)	0 (0)	5 (83.3)	0 (0)	0 (0)
Other, n (%)						
Blood sodium decreased	0 (0)	0 (0)	0 (0)	1 (16.7)	1 (16.7)	0 (0)
GGT increased	0 (0)	0 (0)	0 (0)	1 (16.7)	1 (16.7)	0 (0)
Hyponatremia	1 (16.7)	1 (16.7)	0 (0)	1 (16.7)	1 (16.7)	0 (0)
Hypophosphatemia	0 (0)	0 (0)	0 (0)	1 (16.7)	1 (16.7)	0 (0)
Lipase increased	0 (0)	0 (0)	0 (0)	1 (16.7)	0 (0)	1 (16.7)
Amylase increased	1 (16.7)	0 (0)	1 (16.7)	1 (16.7)	0 (0)	0 (0)
Blood bilirubin increased	0 (0)	0 (0)	0 (0)	1 (16.7)	1 (16.7)	0 (0)
Adrenal insufficiency	1 (16.7)	1 (16.7)	0 (0)	0 (0)	0 (0)	0 (0)
Prolonged QT	4 (66.7)	0 (0)	0 (0)	2 (33.3)	0 (0)	0 (0)
Weight decrease	3 (50.0)	0 (0)	0 (0)	2 (33.3)	0 (0)	0 (0)
Dysgeusia	2 (33.3)	0 (0)	0 (0)	1 (16.7)	0 (0)	0 (0)
Hypoesthesia	2 (33.3)	0 (0)	0 (0)	0 (0)	0 (0)	0 (0)
Fatigue	2 (33.3)	0 (0)	0 (0)	4 (66.7)	0 (0)	0 (0)
Pyrexia	1 (16.7)	0 (0)	0 (0)	3 (50.0)	0 (0)	0 (0)

*G* grade, *GGT* gamma-glutamyltransferase, *ipi* ipilimumab

^a^Only toxicities that reached Grade 3/4 in severity or occurred in >20 % of patients in either cohort are presented

^b^Assessment based on National Cancer Institute Common Terminology Criteria for Adverse Events version 3.0

Table 4 provides a summary of immune-related adverse events (irAEs) in patients who received PTX/CBDCA and at least one dose of ipilimumab. The most commonly reported irAEs across the two study cohorts were grade 1/2 toxicities affecting the skin and subcutaneous tissues (*n* = 5, Cohort A; *n* = 6, Cohort B), gastrointestinal tract (*n* = 4, Cohort A; *n* = 1, Cohort B), and nervous system (*n* = 3, Cohort A; *n* = 5, Cohort B). Two patients in Cohort A experienced a grade 3/4 irAE (*n* = 1, grade 3 adrenal insufficiency; *n* = 1, grade 4 increase in amylase). The grade 3 adrenal insufficiency occurred on Day 159, after 6 cycles of chemotherapy and 4 doses of ipilimumab. It was treated with oral prednisolone that was continued through to the last follow-up assessment due to unresolved decreases in both adrenocorticotropic hormone and cortisol. The grade 4 increase in amylase required no treatment. Two patients in Cohort B experienced a grade 3/4 irAE (*n* = 1, grade 3 enterocolitis, grade 3 increase in blood bilirubin, and grade 4 increase in lipase; *n* = 1, grade 3 increase in gamma-glutamyltransferase [GGT]). The grade 3 enterocolitis resolved with treatment with a high-dose steroid, and the grade 3 increase in blood bilirubin was treated with agents including oral prednisolone, levofloxacin, and meropenem, and parenteral nutrition. The grade 4 increase in lipase and grade 3 increase in GGT both resolved without treatment. One patient in Cohort B experienced an irAE of grade 2 pneumonitis on Day 126 after receiving 5 cycles of chemotherapy and 3 doses of ipilimumab. The pneumonitis resolved following treatment with intravenous methylprednisolone and then oral prednisolone which was gradually tapered.

**Table 4:** Immune-related adverse events in patients who received PTX/CBDCA and at least one dose of ipilimumab

Toxicities^a^	Cohort A			Cohort B		
	(*n* = 6)			(*n* = 6)		
	Ipi 3 mg/kg			Ipi 10 mg/kg		
	G1/2	G3/4	Total	G1/2	G3/4	Total
Skin and subcutaneous tissue disorders	5 (83.3)	0 (0)	5 (83.3)	6 (100.0)	0 (0)	6 (100.0)
Rash	5 (83.3)	0 (0)	5 (83.3)	3 (50.0)	0 (0)	3 (50.0)
Alopecia	1 (16.7)	0 (0)	1 (16.7)	2 (33.3)	0 (0)	2 (33.3)
Pruritus	0 (0)	0 (0)	0 (0)	2 (33.3)	0 (0)	2 (33.3)
Pigmentation disorder	1 (16.7)	0 (0)	1 (16.7)	0 (0)	0 (0)	0 (0)
Dermatitis acneiform	0 (0)	0 (0)	0 (0)	1 (16.7)	0 (0)	1 (16.7)
Rash maculo-papular	0 (0)	0 (0)	0 (0)	1 (16.7)	0 (0)	1 (16.7)
Gastrointestinal disorders	4 (66.7)	0 (0)	4 (66.7)	1 (16.7)	1 (16.7)	2 (33.3)
Diarrhea	3 (50.0)	0 (0)	3 (50.0)	1 (16.7)	0 (0)	1 (16.7)
Gastritis	1 (16.7)	0 (0)	1 (16.7)	0 (0)	0 (0)	0 (0)
Stomatitis	1 (16.7)	0 (0)	1 (16.7)	1 (16.7)	0 (0)	1 (16.7)
Enterocolitis	0 (0)	0 (0)	0 (0)	0 (0)	1 (16.7)	1 (16.7)
Nervous system disorders	3 (50.0)	0 (0)	3 (50.0)	5 (83.3)	0 (0)	5 (83.3)
Hypoesthesia	2 (33.3)	0 (0)	2 (33.3)	0 (0)	0 (0)	0 (0)
Peripheral motor neuropathy	1 (16.7)	0 (0)	1 (16.7)	0 (0)	0 (0)	0 (0)
Peripheral sensory neuropathy	1 (16.7)	0 (0)	1 (16.7)	5 (83.3)	0 (0)	5 (83.3)
Endocrine disorders	1 (16.7)	1 (16.7)	2 (33.3)	0 (0)	0 (0)	0 (0)
Adrenal insufficiency	0 (0)	1 (16.7)	1 (16.7)	0 (0)	0 (0)	0 (0)
Hypoparathyroidism	1 (16.7)	0 (0)	1 (16.7)	0 (0)	0 (0)	0 (0)
Laboratory investigations	0 (0)	1 (16.7)	1 (16.7)	1 (16.7)	2 (33.3)	3 (50.0)
Amylase increased	0 (0)	1 (16.7)	1 (16.7)	1 (16.7)	0 (0)	1 (16.7)
Blood bilirubin increased	0 (0)	0 (0)	0 (0)	0 (0)	1 (16.7)	1 (16.7)
Blood corticotrophin decreased	0 (0)	0 (0)	0 (0)	1 (16.7)	0 (0)	1 (16.7)
Blood cortisol decreased	0 (0)	0 (0)	0 (0)	1 (16.7)	0 (0)	1 (16.7)
Blood TSH decreased	0 (0)	0 (0)	0 (0)	1 (16.7)	0 (0)	1 (16.7)
GGT increased	0 (0)	0 (0)	0 (0)	0 (0)	1 (16.7)	1 (16.7)
Lipase increased	0 (0)	0 (0)	0 (0)	0 (0)	1 (16.7)	1 (16.7)
Respiratory, thoracic and mediastinal disorders	0 (0)	0 (0)	0 (0)	1 (16.7)	0 (0)	1 (16.7)
Pneumonitis	0 (0)	0 (0)	0 (0)	1 (16.7)	0 (0)	1 (16.7)

*G* grade, *GGT* gamma-glutamyltransferase, *ipi* ipilimumab, *TSH* thyroid-stimulating hormone

^a^Assessment based on National Cancer Institute Common Terminology Criteria for Adverse Events version 3.0

### Efficacy data

Efficacy was assessed in patients who received at least one dose of study medication (*N* = 15). In Cohort A (3 mg/kg, *n* = 8), 3 patients achieved a partial response (PR) and an additional 3 patients achieved stable disease (SD). Two patients were not evaluable because they discontinued prior to the first tumor assessment due to chemotherapy toxicity. In Cohort B (10 mg/kg, *n* = 7), 3 patients achieved a PR and an additional 4 patients achieved SD. One patient who achieved SD requested to discontinue prior to the first dose of ipilimumab 10 mg/kg.

### Pharmacokinetic data

**Fig. 2:**
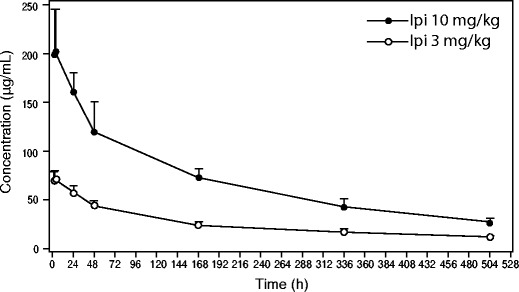
Ipilimumab serum concentration profile versus time during Cycle 3. Open circles, ipilimumab 3 mg/kg plus PTX/CBDCA group (*n* = 6); closed circles, ipilimumab 10 mg/kg plus PTX/CBDCA group (*n* = 6). Data are mean plus standard deviation. On Day 1 of Cycle 3, blood serum samples were drawn at the following time points: Pre-infusion, 1.5 h (just prior to the end of ipilimumab dosing), 4 h and 24 h

Table 5 provides summary statistics for ipilimumab PK parameters during Cycle 3 and PK data from non-Japanese patients treated with ipilimumab in previous clinical studies [9, 10]. Serum ipilimumab levels were elevated with median values of T_max_ from 2.75 to 3.98 h. The observed mean T-HALF of ipilimumab was approximately 11 to 13 days. The geometric mean CL was 12.0 to 14.4 mL/h, and Vss was approximately 5 L. The PK profile in this study was similar to that reported in previous clinical trials of non-Japanese patients [9, 10]. The concentration of serum ipilimumab over time is shown in Fig. 2.

**Table 5:** Summary statistics for ipilimumab pharmacokinetic parameters during Cycle 3 and data from non-Japanese patients in other studies

Dose	C_max_ (μg/mL) Geo. Mean (%CV)	AUC_(0-21d)_ (μg × hr/mL) Geo. Mean (%CV)	T_max_ (h) Median (Min-Max)	T-HALF (day) Mean (SD)	CL (mL/hr) Geo. Mean (%CV)	Vss (L) Geo. Mean (%CV)
Japanese patients (current study)						
Cohort A Ipi 3 mg/kg (*n* = 6)	72.8 (12)	12,632 (12)	2.75 (1.37–4.08)	13.3 (3.64)	12 (17)	4.97 (17)
Cohort B Ipi 10 mg/kg (*n* = 6)	201 (21)	36,489 (21)	3.98 (1.45–23.8)	11.3 (2.83)	14.4 (25)	5.35 (22)
Non-Japanese patients (historical data)						
Ipi 3 mg/kg (*N* = 12)^a^	84.5	12,384	N/A	15.25	N/A	N/A
Ipi 10 mg/kg (*N* = 14)^b^	234.54 (29)	46925.36 (36)	1.51 (1.5-24.0)	13.93 (7.54)	11.63 (50)	5.10 (25)

*AUC* area under the concentration-versus-time curve, *CL* clearance, *C*_*max*_ peak plasma concentration, *CV* coefficient of variation, *ipi* ipilimumab, *N/A* data not available, *SD* standard deviation, *T-HALF* time required to decrease the drug concentration in the blood by half, *Vss* volume of distribution at steady-state

^a^Data from non-Japanese melanoma patients treated with hybridoma-derived ipilimumab 3 mg/kg monotherapy in study MDX010-15 [9]

^b^Data from non-Japanese melanoma patients treated with ipilimumab 10 mg/kg plus paclitaxel and carboplatin in study CA184-078 [10]

### Immunogenicity

A total of 61 samples from the 12 ipilimumab-treated patients were evaluated for the presence of HAHA to ipilimumab; all samples were negative for HAHA.

## Discussion

In this phase I study in Japanese patients with advanced NSCLC using the definition defined in the protocol, and agreed upon by the sponsor and Pl, the recommended dose was identified as 10 mg/kg of ipilimumab in a phased combination with PTX/CBDCA. This dosage of ipilimumab is being studied in two on-going phase III lung cancer studies: CA184-104 (CT.gov, NCT01285609) and CA184-156 (CT.gov, NCT01450761) [11, 12].

Overall, the safety profile was consistent with data from previous studies of ipilimumab in combination with PTX/CBDCA [6] and known toxicities of ipilimumab monotherapy [13] and PTX and CBDCA [14]. Two out of 6 patients treated with ipilimumab 3 mg/kg experienced DLTs with 3 events reported: grade 3 febrile neutropenia, grade 4 increased amylase, and grade 4 thrombocytopenia. The increased amylase required no treatment. Transient febrile neutropenia and thrombocytopenia are common chemotherapy-related AEs [14]. One of 6 patients treated with ipilimumab 10 mg/kg had DLT with 3 events reported: grade 3 enterocolitis, grade 3 increased total bilirubin, and grade 4 increased lipase. Enterocolitis is a common irAE associated with ipilimumab [13]. Increased bilirubin has been described as associated with ipilimumab [13] and chemotherapy [15], and increased lipase is commonly experienced as a chemotherapy-related AE [16] as well. In both dose cohorts, the majority of AEs were hematological, although non-hematological toxicities were also reported (Table 3). There was no clear association between the incidence of AEs and the dose of ipilimumab, as reported in prior studies [17]. Most common safety events associated with ipilimumab are inflammatory in nature, which may reflect its immune-based mechanism of action [18]. The most common irAEs across the two study cohorts were grade 1/2 toxicities affecting the skin, gastrointestinal tract, and nervous system. Immune-related toxicities were manageable with established treatment guidelines [13].

Although conclusions regarding efficacy in phase I studies are necessarily limited, ipilimumab in a phased combination with PTX and CBDCA demonstrated encouraging preliminary antitumor activity. Among the 12 evaluable patients who received at least one dose of ipilimumab, 3 patients in each cohort achieved a partial response, and an additional 3 patients in each cohort achieved stable disease. Stable disease is a potentially clinically relevant outcome with ipilimumab treatment because some patients experience a slow and steady decrease in total tumor burden following a period of SD [19, 20]. Antitumor activity was demonstrated also in the phase II CA184-041 study [6]. Among patients who received phased ipilimumab in combination with PTX and CBDCA (*n* = 68) in study CA184-041, 22 achieved an immune-related partial response and 37 had an immune-related SD, as assessed by a blinded independent radiologic review committee [6, 20].

The PK profile of ipilimumab at two doses, 3 mg/kg and 10 mg/kg, was also evaluated in the current study. Data from previous studies have shown no major PK interactions between ipilimumab administered at 10 mg/kg in Caucasians and PTX/CBDCA [10]. Acknowledging the limitations of comparing data from different studies, the PK characteristics of ipilimumab observed in the current study were similar to those previously reported in clinical trials of non-Japanese patients [9, 10].

Although ipilimumab is a fully human antibody, it has the potential to induce formation of anti-drug antibodies when it is administered as a therapeutic agent [21]. None of the ipilimumab-treated patients in this study had HAHA, and therefore there was no evidence of an anti-ipilimumab antibody response.

In summary, the recommended dose for ipilimumab in combination with PTX/CBDCA in Japanese patients with NSCLC was identified as 10 mg/kg. The first 2 treatment cycles in this study were PTX/CBDCA every 3 weeks and the last 4 treatment cycles were ipilimumab plus PTX/CBDCA every 3 weeks. The safety profile of ipilimumab in combination with PTX and CBDCA was consistent with the previously defined profile established in global clinical studies. Overall, the PK characteristics of ipilimumab in combination with PTX and CBDCA were similar to those demonstrated in global clinical studies in non-Japanese subjects.
